# Fungal Infections in Pediatric Patients With Hematologic Malignancies and Stem Cell Transplantation: Impact on the Upper and Lower Respiratory Systems

**DOI:** 10.1155/cjid/8766717

**Published:** 2026-04-17

**Authors:** Matin Pourghasem, Seyed Ahmad Tabatabaii, Seyedeh Zalfa Modarresi, Abdolhamid Jafari Nodoushan, Nafise Fadavi, Maedeh Soflaee, Ali Hosseini Vajari, Fatemeh Khazaii, Babak Shahhosseini, Korosh Fakhimi Derakhshan, Saeid Sadat Mansouri

**Affiliations:** ^1^ Pediatric Diseases Research Center, Guilan University of Medical Sciences, Rasht, Iran, gums.ac.ir; ^2^ Department of Pediatrics, Mofid Children’s Hospital, Shahid Beheshti University of Medical Sciences, Tehran, Iran, sbmu.ac.ir; ^3^ Children Growth Disorder Research Center, Comprehensive Research Institute for Maternal and Child Health, Shahid Sadoughi University of Medical Sciences, Yazd, Iran, ssu.ac.ir; ^4^ Department of Pediatrics, Tehran Medical Sciences, Islamic Azad University, Tehran, Iran, azad.ac.ir; ^5^ Cardiovascular Research Center, Rajaie Cardiovascular Institute, Tehran, Iran; ^6^ Department of Otolaryngology, Dr. Masih Daneshvari Hospital, Shahid Beheshti University of Medical Sciences, Tehran, Iran, sbmu.ac.ir; ^7^ Department of Pediatrics, Imam Ali Hospital, Alborz University of Medical Sciences, Karaj, Iran, abzums.ac.ir

**Keywords:** antifungal prophylaxis, diagnostic performance, hematologic malignancies, hematopoietic stem cell transplantation, invasive fungal disease, pediatric patients, risk stratification

## Abstract

Invasive fungal infections (IFIs) are a leading cause of morbidity and mortality in children with hematological malignancies as well as those undergoing hematopoietic stem cell transplantation (HSCT). Extreme immunological dysregulation secondary to severe neutropenia, T‐cell lymphopenia, graft‐versus‐host disease (GVHD), intensive chemotherapy regimens, and conditioning therapy for HSCT, as well as primary immunodeficiencies (PIDs), render these patients highly susceptible to both opportunistic and pathogenic fungal infections. Despite advances in antifungal drugs and diagnostic tools, it is very difficult in these children to provide timely diagnosis and optimal management of IFIs because of the nonspecific clinical manifestations, the invasiveness of present diagnostic modalities in pediatric patients, and biomarker kinetics differences in various pediatric age groups, along with a lack of incorporation of immunological–pharmacological maturity–associated variability in the existing scoring systems borrowed from adults. This narrative review provides a comprehensive and contemporary assessment of the epidemiology, host‐related risk factors, clinical presentations, diagnostic criteria, and management practices for IFIs in children with hematological malignancies and following HSCT. It also highlights the role of EORTC/MSGERC criteria in defining IFIs as probable, proven, and possible infections and explores the sensitivity and specificity of noninvasive methods such as the galactomannan index, polymerase chain reaction (PCR), ß‐D‐glucan assay, high‐resolution CT scans (HRCTs), and the latest approaches including next‐generation sequencing (NGS) and metagenomics. This review points out significant gaps in pediatric research studies and supports efforts to optimize healthcare use with risk‐prediction models rather than just relying on current algorithms.

## 1. Introduction

Invasive fungal infections (IFIs) remain a significant concern for immunocompromised children, particularly those with hematologic malignancies or those undergoing hematopoietic stem cell transplantations (HSCTs) [1–3]. Infections occur due to immunocompromised conditions like neutropenia, T‐cell lymphopenia, or graft‐versus‐host disease (GVHD), often secondary to chemotherapy, transplant conditioning or various primary immunodeficiencies (PIDs) [4, 5]. Opportunistic fungi, including *Candida* spp., *Aspergillus* spp., and Mucorales, are generally more pathogenic in the context of neutropenia, while pathogenic fungi, including Cryptococcus, Histoplasma, and Blastomyces, are more likely to cause infection in immunocompromised children with T‐cell lymphopenia [6, 7]. Infections from either of these groups of fungi remain a threat for morbidity and mortality due to diagnostic and therapeutic limitations in children with hematologic malignancies and those undergoing HSCT, although current treatments have shown a significant improvement in survival rates for this group of children [7–13]. In addition, chemotherapy‐related lung infections, which can be managed by using protein nanoparticles, highlight the need for protective strategies due to complications from the malignancy and other associated diseases [14, 15]. Current clinical practice lacks evidence‐based, risk‐stratification tools for pediatrics, leading to substantial practice variation and suboptimal resource allocation. Existing risk‐assessment models primarily derive from adult oncology populations and fail to account for developmental immune system differences, age‐related pharmacokinetic variations, and unique pathogen susceptibility patterns in children [16, 17]. Limited validated, multicenter risk‐prediction models exist specifically for pediatric patients with hematologic malignancies, resulting in challenges with both overtreatment of low‐risk patients and delayed intervention in high‐risk cases.

Furthermore, while noninvasive diagnostic modalities have shown promise, their performance characteristics in pediatric populations require better definition. Systematic evaluation of diagnostic test accuracy with performance metrics in pediatrics remains incomplete, requiring clinicians to extrapolate from adult data with uncertain validity [18, 19]. The economic implications of diagnostic and therapeutic decision‐making in pediatric fungal infections have received limited attention, despite their importance for healthcare policy and resource allocation decisions, particularly in resource‐limited settings where pediatric cancer incidence is rising [20, 21]. Moreover, managing pediatric leukemia is complicated by supportive care challenges, particularly regarding the role of granulocyte transfusion therapy. The clinical efficacy of this therapy remains poorly defined for patients undergoing multiple treatment cycles resulting in persistent neutropenia. While some studies suggest potential benefits, systematic evaluation of this intervention remains limited [13].

**FIGURE 1:**
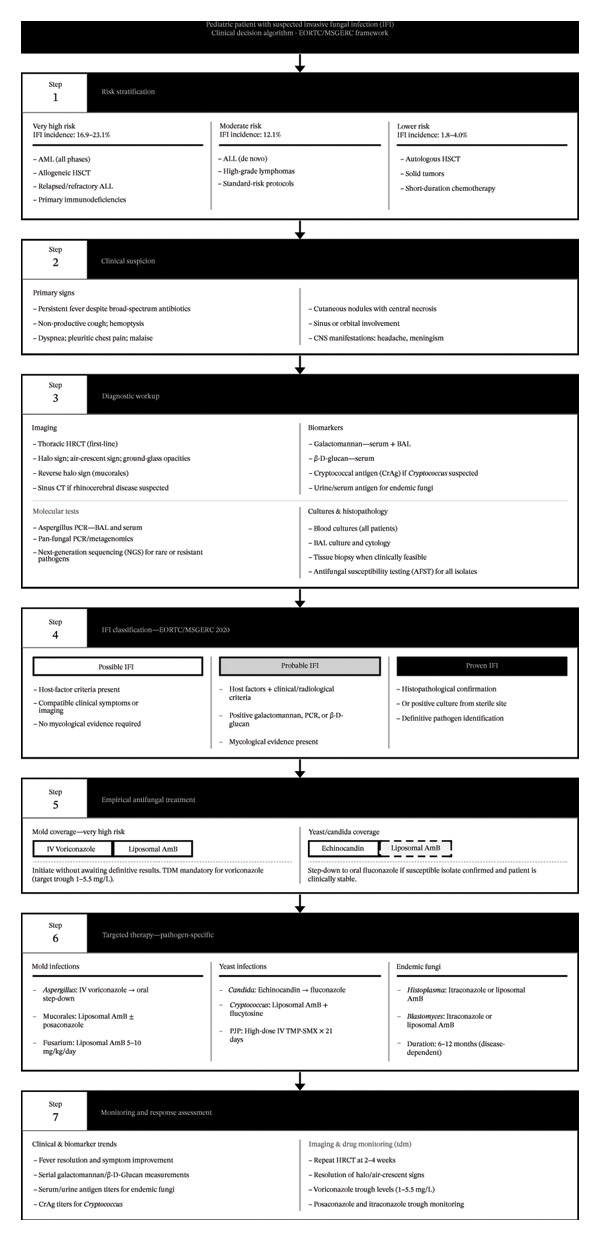
Systematic clinical algorithm for the suspicion, diagnostic workup, classification, and management of invasive fungal infections (IFIs) in pediatric patients with hematologic malignancies or hematopoietic stem cell transplantation, based on EORTC/MSGERC 2020 criteria. Abbreviations: AFST, antifungal susceptibility testing; ALL, acute lymphoblastic leukemia; AML, acute myeloid leukemia; AmB, amphotericin B; BAL, bronchoalveolar lavage; CNS, central nervous system; CrAg, cryptococcal antigen; EORTC/MSGERC, European Organization for Research and Treatment of Cancer/Mycoses Study Group Education and Research Consortium; GGO, ground‐glass opacities; GM, galactomannan; GVHD, graft‐versus‐host disease; HRCT, high‐resolution computed tomography; HSCT, hematopoietic stem cell transplantation; IFI, invasive fungal infection; IV, intravenous; NGS, next‐generation sequencing; PCR, polymerase chain reaction; PJP, *Pneumocystis jirovecii* pneumonia; TDM, therapeutic drug monitoring; TMP–SMX, trimethoprim–sulfamethoxazole.

Recent advances in diagnostic technology, such as polymerase chain reaction (PCR), galactomannan (GM) antigen testing, and imaging studies, have emerged, which have increased sensitivity to detect infections at a young age. Gold‐standard methods, like tissue biopsy, are invasive techniques and are often limited in pediatric studies, leading to reliance on noninvasive techniques with carefully validated performance characteristics [4, 22]. This review presents the epidemiology, presentation, and management approaches of respiratory fungal infections in immunocompromised children with hematologic cancers and transplant recipients, categorized as possible, probable, and proven according to EORTC/MSGERC criteria [23–25]. The current study provides practical guidance in improving the management and quality of life of high‐risk pediatric patients based on evidence‐based risk stratification and management plans tailored to childhood immunodeficiencies, including neutropenia, T‐cell lymphopenia, and combined immunodeficiencies [16, 26, 27]. Figure 1 presents a comprehensive algorithm summarizing the systematic approach to clinical suspicion, diagnostic workup, and classification of IFI in pediatric patients.

## 2. Methods

This research was conducted in compliance with established formats for narrative reviews. Systematic literature searches were performed in major medical databases, such as PubMed, Embase, and the Cochrane Library, exploring a wide range of literature, from historical landmark studies to contemporary publications, on IFIs in children with hematologic malignancies or HSCT.

Search terms consisted of a combination of keywords related to IFIs, the pediatric population, hematologic malignancies, and stem cell transplantation. Preference was given to recent publications, systematic reviews, clinical guidelines, as well as multicenter studies related to the pediatric population.

This study integrates current evidence to develop clinical frameworks, focusing on diagnostics, treatment, and prophylaxis in pediatric hematology–oncology patients.

## 3. Epidemiology

IFIs remain a significant healthcare challenge in immunocompromised children, particularly those with hematologic malignancies such as AML and ALL or those undergoing HSCT [13, 28]. In a 14‐year surveillance conducted among pediatric hematology–oncology departments, there was a fungal infection prevalence of 5.9% in total patients, but this varied according to disease: 23.1% in those with AML, 12.1% in those with ALL, and 1.8% in those classified under solid tumors [28]. In pediatric stem cell transplant recipients, the incidence of fungal infections typically ranges from 3.4% to 3.7%, predominantly manifest as invasive aspergillosis and candidiasis. These infections are frequently a consequence of prolonged neutropenia induced by pretransplant conditioning chemotherapy [17–19]. Despite the implementation of antifungal prophylaxis, breakthrough infections continue to complicate recovery in high‐risk patients. Notably, the incidence varies significantly by transplant type, with allogeneic recipients facing a substantially higher risk (16.9%) than those undergoing autologous transplantation (4.0%) [28]. Data from the Transplant‐Associated Infection Surveillance Network and SEIFEM studies indicate that approximately 49% of these infections are proven cases [19, 20]. T‐cell lymphopenia, often exacerbated by GVHD and intensive immunosuppressive regimens, significantly predisposes patients to IFIs. Community environmental surveillance has demonstrated a close link between the concentration of airborne spores of molds and the risk of infections. Recent findings have also indicated a bidirectional link where chronic GVHD can also increase the risk of fungal infections [29–31]. In hematologic malignancies, fungal infection epidemiology varies by disease type and treatment phase. In ALL, fungal infections contribute to 20% of infection‐related deaths [13, 28]. Children with de novo ALL are at moderate risk, but those with relapsed disease face substantially higher fungal infection risk due to intensified salvage chemotherapy and prolonged immunosuppression, necessitating tailored prophylaxis and monitoring strategies [28, 32].

## 4. Patients at Risk

Host defense against IFIs relies on the innate immune system, mediated by phagocytes such as neutrophils, and the adaptive immune system. Impairments in the number or function of these immune cells significantly increase fungal infection susceptibility in immunocompromised pediatric patients with hematologic conditions [33, 34]. Recent clinical evidence and multicenter prospective studies have identified high‐risk pediatric populations by establishing independent risk factors to develop robust predictive models for invasive fungal diseases [35].

### 4.1. PIDs Associated With Hematologic Malignancies

PIDs play a critical role in augmenting susceptibility to IFDs when predisposing children to, or being associated with, hematologic malignancies. Severe combined immunodeficiency (SCID), often necessitating HSCT as a definitive treatment, places patients at an increased risk for fungal infections during the transplant process. Chronic granulomatous disease (CGD), characterized by impaired granulocyte function, increases the risk for both fungal infections and secondary hematologic malignancies. Similarly, congenital neutropenia syndromes, which primarily deplete neutrophil counts, may progress to myelodysplastic syndrome or acute leukemia; these require intensive chemotherapy that further exacerbates infection risk [2, 4, 5].

### 4.2. Hematologic Malignancy

#### 4.2.1. Acute Myeloid Leukemia

Intensive induction and consolidation chemotherapy regimens for AML induce prolonged and profound neutropenia, coupled with significant T‐cell dysfunction, leading to an increased risk for fungal infections. In pediatric patients with AML, having an absolute neutrophil count of 500 cells/μL or less at the beginning of a chemotherapy cycle has been shown to triple the risk of developing invasive fungal disease [13, 36–38].

#### 4.2.2. Acute Lymphoblastic Leukemia

Children with de novo ALL face a moderate risk of fungal infections during standard therapy, with a prevalence of 12.1% primarily driven by *Pneumocystis jirovecii*, while patients with relapsed or refractory disease face substantially higher risks due to intensified salvage regimens and prolonged immunosuppression from prior exposure to multiple chemotherapeutic agents. Key risk factors during induction therapy for childhood ALL include moderate neutropenia, T‐cell lymphopenia, and mucositis related to chemotherapy [28, 39, 40].

#### 4.2.3. Lymphomas

Children with high‐grade lymphomas undergoing intensive chemotherapy protocols, particularly those requiring salvage regimens or autologous transplantation, face neutropenia and lymphocyte dysfunction, leading to increased susceptibility to fungal infections [4, 13].

### 4.3. HSCT

#### 4.3.1. Pre‐Engraftment Phase

Myeloablative conditioning regimens result in prolonged and profound neutropenia, compromising innate immune function and causing increased risk for invasive aspergillosis and candidiasis. The duration of postconditioning neutropenia varies based on factors such as stem cell source and dose but typically persists for several weeks before engraftment [4, 13].

#### 4.3.2. Postengraftment Phase

Following the resolution of neutropenia via neutrophil engraftment, the risk of IFDs is predominantly driven by T‐cell dysfunction. This impairment, which can persist for several months post‐transplant, is attributable to delayed immune reconstitution, intensive immunosuppressive therapies, and the prolonged use of calcineurin inhibitors [4, 13].

#### 4.3.3. Late Phase

**FIGURE 2:**
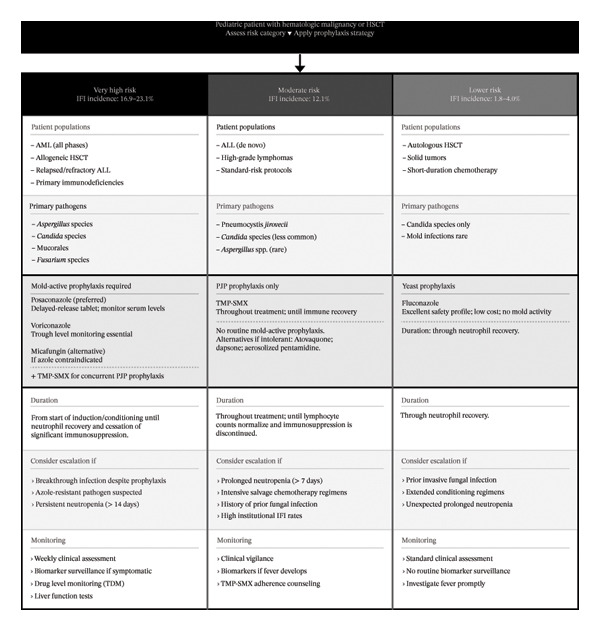
Risk‐stratified antifungal prophylaxis framework for pediatric patients with hematologic malignancies and hematopoietic stem cell transplantation recipients, including recommended agents, duration, escalation criteria, and monitoring parameters. Abbreviations: ALL, acute lymphoblastic leukemia; AML, acute myeloid leukemia; HSCT, hematopoietic stem cell transplantation; IFI, invasive fungal infection; PJP, *Pneumocystis jirovecii* pneumonia; TDM, therapeutic drug monitoring; TMP–SMX, trimethoprim–sulfamethoxazole.

Patients with chronic GVHD (cGVHD) on prolonged immunosuppression face a heightened vulnerability to opportunistic and encapsulated fungal pathogens. This elevated risk results from a synergistic effect of the underlying T‐cell dysfunction and the multifaceted suppressive therapies required for disease management [4, 13]. A risk‐based decision‐making framework for pediatric patients is illustrated in Figure 2.

## 5. Diagnostic Standards

The diagnosis of IFI in pediatric patients is categorized as possible, probable, or proven based on the European Organization for Research and Treatment of Cancer and Mycoses Study Group Education and Research Consortium (EORTC/MSGERC) consensus criteria, which integrate clinical, radiological, and microbiological findings [22, 41]. Tissue biopsy remains the gold standard for definitive diagnosis, but its invasiveness limits its application, particularly in critically ill children. As a result, noninvasive diagnostic methods, such as GM antigen detection, PCR, and imaging, are widely used, with sensitivity and specificity varying by patient immune status and pathogen type [4, 22] (Table 1).

**TABLE 1:** Performance characteristics of noninvasive diagnostic tests in pediatric IFI.

Diagnostic test	Specimen type	Sensitivity (%)	Specificity (%)	Advantages	Limitations
Galactomannan (GM)	BAL	56%–82% [42]	85%–98% [42]	Higher sensitivity than serum GM due to greater airway fungal burden; useful in suspected pulmonary aspergillosis [43].	False positives with β‐lactams; reduced sensitivity after antifungal exposure [43].
Galactomannan (GM)	Serum (Screening)	0%–100% [13]	50%–100% [13]	Noninvasive; may allow earlier detection in high‐risk hematology patients; useful for serial monitoring [42, 43].	Highly variable performance in children; sensitivity decreases with antifungal use [42]
Galactomannan (GM)	Serum (Diagnostic use)	14%–100% [13]	35%–100% [13]	Incorporated as microbiological criterion in EORTC/MSG definitions; widely available [43].	Cross‐reactivity; considered an unreliable surveillance test in some pediatric guidelines [43]
PCR (*Aspergillus*)	BAL	88.6% [42]	95.5% [42]	High sensitivity; may outperform culture; quantitative fungal load may correlate with prognosis [42].	Lack of assay standardization; interlaboratory variability; often species‐targeted (e.g., *A. fumigatus*) [42].
PCR (*Aspergillus*)	Serum/Various	0%–100% [13]	36%–83% [13]	Rapid detection; higher diagnostic yield than culture in some studies [13].	Variable specificity in children; not universally recommended for routine screening [13].
β‐D‐Glucan (BDG)	Serum	Variable [13]	Variable [13]	Broad‐spectrum fungal detection (*Aspergillus*, *Candida*, *Fusarium*) [13].	False positives with blood products, IVIG, hemodialysis membranes; typically negative in mucormycosis; limited pediatric validation [13].
Blood Culture	Blood	Low for molds; moderate‐high for yeasts [13]	High [13]	Gold standard for candidemia; enables species identification and susceptibility testing [13].	Very low sensitivity for filamentous fungi; prolonged turnaround time [13].
Next‐Generation Sequencing (NGS)	BAL, Tissue, Plasma	96.6% [13]	98.2% [13]	Detects rare or uncultivable pathogens; useful in diagnostically challenging cases [13].	High cost; complex bioinformatics; limited pediatric‐specific validation; potential contamination issues [13].

*Note:* Reported sensitivity and specificity ranges reflect pooled data from pediatric and mixed pediatric–adult studies. Actual performance may vary depending on the specimen type, assay cutoff values, antifungal exposure, underlying immune status, and concurrent medications such as β‐lactam antibiotics. BAL, bronchoalveolar lavage.

Abbreviations: IFI, invasive fungal infection; PCR, polymerase chain reaction.

### 5.1. GM Assay

The GM test is variably sensitive depending on the sample type and patient population. In pediatric cases, it has demonstrated a sensitivity of 50%–82% for BAL fluid and serum in neutropenic patients, with specificity rates of 80%–95% [42, 43]. Positive predictive values vary with the prevalence of disease, but the negative predictive value remains high in the appropriate patient subset. False‐positive test results may occur due to cross‐reactivity with beta‐lactam antibiotics (such as piperacillin–tazobactam), other fungal pathogens (like Histoplasma), food antigens, or coadministered IV medications [43].

### 5.2. PCR

Real‐time PCR has proven more effective for *Aspergillus* species in BAL fluid specimens compared to conventional culture. Pan‐fungal PCR platforms are also useful in wide‐spectrum pathogen detection, although coinfections are potential sources of complexity [42]. Studies report diagnostic yields of 63%–96% for invasive mold infections using PCR testing [13].

### 5.3. Beta‐D‐Glucan (BDG)

This biomarker demonstrates moderate diagnostic utility, as its performance varies depending on the causative organism. Adult trials with hematologic malignancy have established high levels of specificity and positive predictive value, especially if two test results are positive, although experience in children is limited and the appropriate cutoff levels in children remain undefined [13]. It is worth noting that the results of BDG tests are mostly negative in mucormycosis infections. False‐positive results can also occur due to bacterial infections, surgery, or hemodialysis [43].

### 5.4. Emerging Diagnostic Technologies

Next‐generation sequencing (NGS) and metagenomic technologies are emerging as powerful diagnostic tools, offering superior sensitivity for identifying rare or fastidious pathogens such as Fusarium spp. in pediatric BAL samples. Beyond detecting organisms missed by conventional cultures, these molecular techniques can identify antimicrobial resistance genes and characterize noncultivatable pathogens previously only detectable via histopathology [13]. Standardization in the pediatric population is, however, difficult owing to differences in the immune status of the patients, possibly smaller sample volumes compared to adult patients, and the need for advanced biotechnology analysis [44, 45].

### 5.5. Imaging Modalities

Thoracic high‐resolution computed tomography (HRCT) remains an important diagnostic tool for IFIs, offering high sensitivity in detecting pulmonary infiltrates and nodules. However, its clinical utility is often hampered by a lack of specificity, as radiological findings frequently overlap with other infectious and noninfectious etiologies. Classic hallmarks, such as the halo and air‐crescent signs, carry a significant prognostic value, particularly in neutropenic populations; nonetheless, these signs are documented less frequently in pediatric cohorts compared to adult patients [46–48].

This diagnostic complexity highlights the urgent need for more specific, noninvasive imaging biomarkers. Recent breakthroughs in molecular imaging, such as immuno‐PET and advanced MRI protocols (e.g., CryptoCEST), are emerging to bridge this gap, enabling the differentiation of fungal pathogens from host inflammatory responses [49]. Consequently, a reliable diagnostic gold standard necessitates a multimodal approach, integrating advanced imaging with biomarkers like GM and PCR to enhance accuracy while minimizing the need for invasive procedures [43, 46–49].

Diagnostic challenges in pediatrics stem from limited sample volumes, age‐related variations in biomarker kinetics, and the risks associated with invasive procedures in critically ill children. Emerging evidence suggests that multibiomarker approaches may reduce the necessity for invasive testing without compromising diagnostic accuracy compared to tissue‐based methods. Consequently, future research should prioritize validating age‐specific criteria for biomarkers and diagnostic platforms in pediatric populations [4, 22].

## 6. Main Respiratory Fungal Pathogens

### 6.1. *Aspergillus fumigatus*

#### 6.1.1. Epidemiology and Pathogenesis

*Aspergillus fumigatus* is the most common opportunistic pathogen among pediatric patients with hematological malignancies and HSCTs [50]. In the environment, its spores convert into an invasive hyphal form upon inhalation by susceptible individuals, including those with persistent neutropenia, impaired granulocyte function, T cell lymphopenia, or GVHD, leading to pulmonary aspergillosis [7, 51]. Invasive molds, particularly *Aspergillus* species, are the primary cause of invasive aspergillosis, with *Aspergillus fumigatus* being the most frequently identified pathogen [13, 52].

#### 6.1.2. Clinical Manifestations

Invasive pulmonary aspergillosis (IPA) begins with nonspecific symptoms that may resemble those of bacterial pneumonia, including persistent fever despite broad‐spectrum antibiotics, nonproductive cough, and malaise [13]. In neutropenic individuals, the disease may progress rapidly to pleuritic chest pain, hemoptysis, and dyspnea. Extrapulmonary dissemination also occurs in a proportion of patients; such disseminated infections predominantly affect the central nervous system (CNS), skin, liver, and kidneys, which serves as a poor prognostic indicator [4, 50]. Invasive aspergillosis can be subdivided into two pathological categories depending on the nature and extent of the infection: Angioinvasive disease, which results from the invasion of hyphae into the pulmonary blood vessels leading to thrombosis and necrosis; and airway‐invasive disease, which manifests as necrotizing tracheobronchitis [4, 13, 53].

#### 6.1.3. Diagnostic Approach

IPA is diagnosed according to the EORTC/MSGERC consensus criteria, which categorize cases into possible, probable, or proven. A possible diagnosis is based on clinical symptoms (e.g., fever, cough) and characteristic radiological findings such as nodules or the halo sign, although these may be less frequent in pediatric cases compared to adults [4, 13]. Probable cases require mycological evidence, traditionally through GM or PCR; however, recent updates emphasize the integration of novel lateral flow assays (LFAs) for rapid detection [54]. A proven case remains dependent on definitive histopathology or positive cultures from sterile sites [22, 41]. While GM reliability is notably higher in neutropenic patients, its sensitivity in non‐neutropenic cohorts can be optimized by combining it with *Aspergillus*‐specific IgG testing [54]. False‐positive results remain a challenge due to cross‐reactivity with beta‐lactam antibiotics or dietary factors [43]. Given that tissue biopsy is often infeasible in pediatric patients and BAL culture sensitivity is limited (35%–63%), the implementation of rapid molecular methods like PCR and point‐of‐care LFA has become crucial for improving early diagnostic accuracy [54].

#### 6.1.4. Radiological Features

IPA presents with key radiological features that aid in diagnosis, though these depend on the host’s immune status [55–57]. In neutropenic patients, the halo sign may progress into an air‐crescent sign, reflecting angioinvasive disease [4, 51, 55]; furthermore, the hypodense sign is now recognized as a highly specific early indicator of this process [57]. These features help guide antifungal therapy and estimate prognosis, although they are observed less frequently in children than in adults, with classic signs like the halo sign appearing in only 11% of pediatric cases [46–49, 56]. In airway‐invasive aspergillosis, radiological findings include lobar or patchy parenchymal consolidation, centrilobular nodules, ground‐glass opacities (GGOs) (often in a tree‐in‐bud pattern), and occasionally, pseudomembranous tracheobronchitis or tracheal necrosis [55, 58].

#### 6.1.5. Treatment Strategy

##### 6.1.5.1. Prophylaxis

Antifungal prophylaxis with mold‐active agents is the cornerstone of prevention in high‐risk pediatric populations, particularly those with AML undergoing intensive chemotherapy or in the pre‐engraftment phase following HSCT. This strategy significantly reduces the incidence of IPA compared to placebo or fluconazole, as the latter provides no coverage against molds [59, 60].

##### 6.1.5.2. Empirical Therapy

As soon as there is clinical and radiological suspicion of IPA, empirical antifungal treatment should be initiated without awaiting definitive results. First‐line empirical therapy typically includes IV voriconazole or liposomal amphotericin B, depending on patient tolerance and institutional protocols [60–62].

##### 6.1.5.3. Targeted Therapy

Following mycological confirmation of *Aspergillus* infection, therapy should be tailored to the specific pathogen. IV Voriconazole is the preferred agent for initial targeted therapy in susceptible strains, providing proven survival benefits and lower toxicity compared to amphotericin B deoxycholate [13]. Transitioning to an oral step‐down regimen is appropriate after clinical stabilization. Diligent therapeutic drug monitoring (TDM) is essential to maintain voriconazole trough levels within the therapeutic range, maximizing efficacy while minimizing hepatotoxicity and neurological adverse effects [60–62].

##### 6.1.5.4. Salvage Therapy

For patients experiencing treatment failure or infection with drug‐resistant organisms, second‐line options include liposomal amphotericin B, posaconazole, or combination therapy with an echinocandin. These combinations may exert a synergistic effect in patients with refractory infections, though they necessitate close monitoring for cumulative toxicities [60–63]. In patients with hematologic malignancies and transplant recipients, the combination of voriconazole and anidulafungin has shown a reduction in mortality rates compared to voriconazole monotherapy, although this finding was at the margin of statistical significance [13, 60, 64].

#### 6.1.6. Treatment Monitoring and Resistance

The emergence of azole‐resistant strains of *Aspergillus fumigatus* poses an increasing clinical challenge. Multi‐ and pan‐azole‐resistant organisms have been identified globally; such resistance may be acquired via environmental exposure, as resistant strains have been isolated even from azole‐naïve patients [13]. Antifungal susceptibility testing (AFST) should be performed routinely. Notably, the prevalence of azole‐resistant *A. fumigatus* strains varies considerably by geographic region, with particularly high rates reported in parts of Europe, Asia, and the Middle East [65]. CNS involvement requires a multifaceted management approach, utilizing antifungal agents with high CNS penetration, comprehensive neurological assessment, and cerebrospinal fluid (CSF) evaluation [13, 60, 62, 64, 65].

### 6.2. *Candida albicans*

#### 6.2.1. Epidemiology and Clinical Significance

*Candida albicans* is a commensal organism that colonizes human skin and mucosa. It remains the most prevalent cause of invasive candidiasis in immunocompromised pediatric patients [66, 67]. Prolonged neutropenia and T‐cell lymphopenia confer high susceptibility to life‐threatening infections, such as pulmonary candidiasis. As noted in a comprehensive review, data from multicenter prospective surveillance of pediatric candidemia indicate that while *Candida albicans* remains the most frequently isolated species, non‐albicans *Candida* species are becoming increasingly prevalent worldwide [13].

#### 6.2.2. Pathogenesis and Risk Factors

*Candida albicans* transitions from a harmless commensal to an invasive pathogen when host defenses are impaired, particularly through the dysfunction of neutrophils and cell‐mediated immunity. Major risk factors in pediatric patients with hematologic malignancies include the administration of broad‐spectrum antibiotics, chemotherapy‐induced mucositis, the presence of central venous catheters (CVCs), total parenteral nutrition (TPN), and glucocorticoid therapy. The pathogenicity of *C. albicans* is significantly enhanced by its ability to form biofilms on medical devices and its capacity to undergo a morphologic transition between yeast and hyphal forms [66–68].

#### 6.2.3. Clinical Manifestations

The clinical presentation of invasive candidiasis includes persistent fever unresponsive to antibiotics, productive cough, pleuritic chest pain, and tachypnea [69]. Pulmonary involvement typically occurs either as primary bronchopneumonia, which often follows aspiration of oropharyngeal secretions in the context of esophageal candidiasis, or as secondary pneumonia resulting from hematogenous spread from gastrointestinal or cutaneous sites. These latter two pathways are more prevalent in immunocompromised hosts. Invasive infection is often diagnosed following the isolation of *Candida* from blood cultures, particularly after intestinal mucosal barrier disruption in neutropenic individuals or through a central catheter [13]. Disseminated infection can involve multiple organs, including the liver, spleen, kidneys, and CNS, which complicates management and is associated with poor outcomes [66].

#### 6.2.4. Diagnostic Approach

The diagnosis of invasive candidiasis is based on the EORTC/MSGERC criteria and is classified into three distinct categories. A possible diagnosis is defined by the presence of clinical manifestations accompanied by nonspecific radiographic changes. A probable diagnosis is established when clinical or radiographic evidence is supported by positive cultures or BDG tests. Finally, a proven diagnosis requires histopathological confirmation of yeast within the lung tissue [22, 41]. Regarding imaging, radiographic findings often include patchy bilateral consolidation with air bronchograms in primary pneumonia, whereas secondary pneumonia is characterized by miliary or larger nodules along with consolidation [66, 70, 71].

While blood culture remains the gold standard for diagnosing candidemia, its clinical utility is often limited by a significant time lag before results become positive [13]. BDG assay is a valuable tool for identifying invasive candidiasis; however, its specificity can be compromised by false‐positive results associated with certain bacterial infections or specific medications. In contrast, modern molecular techniques, including multiplex PCR panels and metagenomic next‐generation sequencing (mNGS), allow for the rapid identification of *Candida* species and detection of azole or echinocandin resistance genes, with superior turnaround time compared to conventional culture methods. Although tissue biopsy remains the gold standard for definitive diagnosis, its clinical application in pediatric populations is limited by its invasive nature and the potential for confounding results when samples are obtained from nonsterile sites [70].

#### 6.2.5. Treatment

Echinocandins, such as caspofungin or micafungin, are recommended as first‐line therapy for invasive candidiasis in pediatric patients due to their potent fungicidal activity and favorable safety profiles, characterized by minimal drug–drug interactions. Liposomal amphotericin B (3–5 mg/kg) serves as a robust alternative, although it necessitates rigorous monitoring for potential nephrotoxicity [70, 72]. For clinically stable patients infected with susceptible *C. albicans* isolates, step‐down therapy to fluconazole is appropriate, particularly to facilitate oral transition and hospital discharge. Treatment should be maintained for at least 14 days following the first negative blood culture, with extended duration required in cases of persistent neutropenia. In patients refractory to primary treatment, management strategies involve switching to a different antifungal class or considering combination therapy [70, 72].

#### 6.2.6. Antifungal Resistance and Prophylaxis

Azole resistance, particularly among non‐albicans *Candida* species, is an emerging concern in pediatric hematology–oncology. Consequently, AFST should be routinely performed for all invasive isolates to guide targeted therapy. While prophylaxis with fluconazole is highly effective in preventing invasive candidiasis in high‐risk children, its use may contribute to a selective shift toward non‐albicans strains with reduced azole susceptibility. Prophylaxis is typically continued until absolute neutrophil count recovery or the resolution of the underlying immunosuppressive condition [73].

#### 6.2.7. Clinical Outcomes

Early diagnosis and the prompt initiation of appropriate antifungal therapy significantly improve survival rates, although mortality remains high in cases of disseminated infection. Comprehensive prevention and early diagnostic strategies are crucial for improving outcomes in pediatric hematology patients [65, 73].

### 6.3. *Pneumocystis jirovecii*

#### 6.3.1. Epidemiology and Risk Factors

*Pneumocystis jirovecii* pneumonia (PJP) is a leading cause of morbidity and mortality in immunosuppressed children [5, 74, 75]. This opportunistic pathogen primarily targets children with impaired cell‐mediated immunity, such as those with ALL or GVHD [76]. Trimethoprim–sulfamethoxazole (TMP–SMX) prophylaxis has significantly decreased the incidence of PJP in ALL and transplant recipients to nearly undetectable levels; however, cases of breakthrough infections due to noncompliance or emerging resistance are being reported [74, 76].

#### 6.3.2. Clinical Presentation

The clinical manifestations of PJP pneumonia can vary, ranging from asymptomatic infections identified incidentally during screening to severe respiratory distress. Common symptoms include fever, nonproductive cough, hemoptysis, chest pain, and dyspnea. Constitutional signs may include weight loss, night sweats, and malaise. In severely immunocompromised patients, the disease may rapidly progress to pleuritic chest pain and cyanosis [4, 76, 77].

#### 6.3.3. Radiological Features

HRCT is the modality of choice, typically demonstrating GGOs in the upper lobes, often with peripheral sparing and interlobular septal thickening. However, these features are not pathognomonic for this infection [4]. Subpleural cysts and pneumatoceles may also be observed, which can lead to spontaneous pneumothorax. Notably, chest radiographs may appear normal in a substantial proportion of patients despite severe clinical symptoms, highlighting the importance of HRCT for early diagnosis [78, 79].

#### 6.3.4. Diagnostic Approach

According to the EORTC/MSGERC criteria, PJP pneumonia is categorized into three distinct levels: possible cases, which involve clinical features and nonspecific radiographic abnormalities such as GGO; probable cases, which require a positive PCR from BAL fluid in association with clinical or radiographic abnormalities; and proven cases, established through histopathological or microscopic evidence of *P. jirovecii* within lung tissue [22, 41]. While tissue biopsy is generally avoided in pediatric populations, a negative BAL PCR result effectively rules out PJP in febrile neutropenic patients [80, 81].

Quantitative PCR (qPCR) offers the highest sensitivity and specificity, with the best diagnostic yields achieved using BAL specimens. However, positive PCR results must be integrated with the clinical presentation, as the test may detect colonization or low‐level exposure in the absence of active disease. Levels of BDG are usually elevated during active PJP infection, but this marker is not specific and may remain elevated even during successful treatment [74].

#### 6.3.5. Treatment Protocols

For *P. jirovecii* pneumonia, first‐line treatment consists of high‐dose intravenous TMP–SMX (trimethoprim 15–20 mg/kg/day and sulfamethoxazole 75–100 mg/kg/day) for 21 days, adjusted according to the severity of the disease [74, 82]. However, the use of adjunctive corticosteroids in children with hematologic malignancies remains controversial [80]. Second‐line therapies include atovaquone, clindamycin–primaquine, intravenous pentamidine, or a combination of dapsone and trimethoprim; all are typically administered for 3 weeks, although clinical evidence for these alternatives in pediatric oncology is limited [82].

#### 6.3.6. Prophylaxis and Resistance

Prophylaxis with TMP–SMX is strongly recommended for high‐risk pediatric patients and should be continued until lymphocyte counts normalize and immunosuppressive medications are discontinued. Alternative prophylactic agents include atovaquone, dapsone, or aerosolized pentamidine, though these may be less effective than TMP–SMX. While primary resistance to TMP–SMX remains rare, it has been reported in certain centers. Breakthrough infections may occur in high‐risk patients due to nonadherence, malabsorption, or drug interactions that reduce therapeutic levels of TMP–SMX, as well as the potential development of resistance. These challenges underscore the need for monitoring compliance and considering alternative prophylaxis in selected high‐risk populations [73].

#### 6.3.7. Clinical Outcomes

Prompt diagnosis and effective therapeutic management have significantly decreased the mortality rate of *Pneumocystis* pneumonia in children with hematological malignancies. However, mortality remains high in severe cases requiring mechanical ventilation, highlighting the critical role of early intervention and vigilant clinical management [74, 82].

### 6.4. Mucormycosis

#### 6.4.1. Epidemiology and Risk Factors

Mucormycosis is the second most common opportunistic mold infection among immunocompromised pediatric patients, primarily associated with severe neutropenia or GVHD [83–85]. It is a rapidly progressive and often fatal infection, particularly in individuals with profound T‐cell immunity defects, active GVHD requiring high‐dose corticosteroid therapy, or prolonged neutropenia. Additional risk factors include iron overload, diabetic ketoacidosis, and nutritional deficiencies. Notably, prior voriconazole prophylaxis may predispose patients to Mucorales colonization, as this agent lacks activity against this fungal order [84–86]. Recent epidemiological updates indicate a significant rise in global incidence during the postpandemic era, primarily driven by uncontrolled diabetes and the widespread use of corticosteroids [87].

#### 6.4.2. Pathogenesis and Clinical Manifestations

Mucorales exhibit profound angioinvasive properties, leading to extensive thrombosis, tissue infarction, and hemorrhagic necrosis within hours of infection onset. The virulence of these pathogens is significantly enhanced by the secretion of mucoricin, a recently identified ricin‐like toxin that blocks protein synthesis and causes vascular leak. Furthermore, the fungal CotH invasins facilitate tissue destruction by specifically binding to host GRP78 receptors, a process exacerbated by hyperglycemia and metabolic acidosis [85]. Pulmonary symptoms often mimic those of IPA, including cough, dyspnea, chest discomfort, and hemoptysis [13, 83, 84]. Rhinocerebral mucormycosis involves the paranasal sinuses and frequently extends to orbital or cerebral tissues. Disseminated disease occurs in a substantial proportion of patients and carries a very poor prognosis [83, 84].

#### 6.4.3. Diagnostic Approach

The diagnosis of mucormycosis is based on the EORTC/MSGERC criteria [22, 41]. HRCT findings may include multiple nodules, wedge‐shaped consolidation typical for pulmonary infarctions, cavitary lesions, or the reverse halo sign [46, 85]. While tissue biopsy remains the gold standard, its use is often limited by its invasive nature. Newer diagnostic frontiers, such as mNGS on BAL fluid, have shown high sensitivity, potentially reaching 81.3%. Additionally, noninvasive breath‐based metabolomics to detect specific volatile organic compounds (VOCs) are emerging as promising tools for earlier detection. Molecular techniques, such as real‐time PCR, show promise but currently lack international standardization [85].

#### 6.4.4. Treatment Approach

The standard treatment for mucormycosis includes high‐dose liposomal amphotericin B (5–10 mg/kg/day). Crucially, voriconazole has no activity against Mucorales; in patients who develop mucormycosis while receiving voriconazole prophylaxis, immediate switch to a Mucorales‐active agent is mandatory. Current guidelines have expanded to include isavuconazole and posaconazole as established first‐line or salvage therapy options. Prompt initiation of treatment upon clinical suspicion is critical. Combination regimens are often considered for refractory cases or infections involving the CNS. For localized infections, aggressive surgical debridement of necrotic tissue plays a pivotal role. Successful management increasingly necessitates a multidisciplinary team (MDT) approach to coordinate complex surgical and medical interventions. Step‐down therapy to oral posaconazole is appropriate for stabilized patients showing a clinical response, requiring diligent TDM [83, 84].

#### 6.4.5. Prognosis

Except for posaconazole and isavuconazole, Mucorales are intrinsically resistant to most azoles and all echinocandins. Although rare, resistance to amphotericin B has been documented and is associated with an extremely poor prognosis. The timing of intervention is critical; delaying Mucorales‐active therapy beyond 5 days from the onset of symptoms has been shown to double the 12‐week mortality rate [83, 84]. The emerging prevalence of uncommon species with varying antifungal susceptibility profiles underscores the necessity for species‐level identification and susceptibility testing. Mucormycosis continues to have a higher mortality rate compared to other IFIs. Early diagnosis and rapid intervention are the most significant predictors of survival in high‐risk pediatric populations [83, 84].

### 6.5. Histoplasma

#### 6.5.1. Epidemiology and Geographic Distribution

Histoplasma capsulatum is an uncommon but severe opportunistic dimorphic fungus [13]. In transplant recipients, it typically manifests at a mean of 5–18 months post‐transplantation. Mortality rates have been reported as high as 67% in allogeneic HSCT recipients [88]. Upon inhalation, the environmental spores convert to a pathogenic yeast phase at body temperature, exhibiting increased virulence in individuals with impaired cell‐mediated immunity. Primary risk factors for pediatric patients include prolonged neutropenia, T‐cell dysfunction, prolonged corticosteroid therapy, and active GVHD requiring aggressive immunosuppression. Disseminated histoplasmosis is associated with exceptionally high mortality rates among severely immunocompromised individuals [13, 88, 89].

#### 6.5.2. Clinical Manifestations

Pulmonary histoplasmosis may present as an initial manifestation of hematologic malignancy or develop following the initiation of immunosuppressive therapy. Symptoms include acute chest discomfort, a dry cough that may progress to a productive cough, low‐grade fever, night sweats, and occasionally broncholithiasis. Disseminated histoplasmosis is common in immunocompromised children, often presenting as fever of unknown origin (FUO), hepatosplenomegaly, lymphadenopathy, and pancytopenia, accompanied by constitutional symptoms such as weight loss and failure to thrive (FTT). Skin lesions, when present, can be a valuable diagnostic clue. CNS involvement typically manifests as chronic meningitis, characterized by headaches, altered mental status, and focal neurological deficits [13, 88, 89].

#### 6.5.3. Diagnostic Approach

Diagnosis of histoplasmosis follows the EORTC/MSGERC criteria: possible, characterized by clinical symptoms such as a dry cough and nonspecific radiological findings; probable, involving positive antigen tests, serology, or culture associated with clinical or radiological evidence; and proven, requiring the isolation of the organism or histopathological evidence within lung or other tissues [22, 41]. Detection of histoplasma capsulatum antigen in urine and serum offers high sensitivity and is the most rapid diagnostic method, particularly in disseminated disease, though its sensitivity is lower in isolated pulmonary involvement. Cross‐reactivity with other endemic fungal infections (such as Blastomycosis or Coccidioidomycosis) may occur. Complement fixation and immunodiffusion tests show variable sensitivity. While culture is definitive, it requires extended incubation periods and specialized mycological expertise. Histopathological examination remains a crucial diagnostic tool; tissue biopsy with specialized staining (e.g., GMS or PAS) characteristic yeast forms within histiocytes. Radiological findings are generally nonspecific, often demonstrating diffuse infiltrates or miliary nodules. Although tissue biopsy and culture remain the gold standards, their invasive nature often necessitates a reliance on antigen detection or serological assays [90–92].

#### 6.5.4. Treatment

##### 6.5.4.1. Mild to Moderate Disease

For localized pulmonary histoplasmosis in stable patients, itraconazole is the drug of choice. The treatment course typically lasts 6–12 months or until clinical and radiological resolution is achieved. Additionally, TDM for itraconazole is strongly advisable to ensure efficacy and minimize toxicity [13, 90, 92].

##### 6.5.4.2. Severe or Disseminated Disease

For severe, disseminated, or CNS‐involved histoplasmosis, as well as for critically ill hospitalized patients, liposomal amphotericin B (3–5 mg/kg/day) is the treatment of choice. Induction therapy is usually administered for 1–2 weeks until clinical stabilization occurs. Following initial recovery, therapy is transitioned or stepped down to itraconazole for a minimum of 12 months or until the resolution of immunosuppression. In cases of histoplasmosis with respiratory distress, a short course of systemic glucocorticoids may be added to mitigate inflammatory complications. Alternatively, voriconazole or posaconazole may be considered for patients who are intolerant to or have contraindications for itraconazole [13, 92].

#### 6.5.5. Prophylaxis and Monitoring

Primary prophylaxis with itraconazole is recommended for high‐risk patients in endemic regions, particularly HSCT recipients with severe GVHD. However, long‐term chronic prophylaxis is generally discouraged due to potential drug interactions, cumulative toxicity, and the lack of standardized universal guidelines. Evaluation of the treatment response relies on a combination of clinical improvement, radiographic clearing, and a significant reduction in serum or urinary antigen levels. While antigen levels typically decrease with effective therapy, they may remain positive for several months despite clinical success; therefore, antigen persistence alone should not be the sole factor in determining the termination of therapy. With appropriate antifungal management, survival rates for disseminated disease have significantly improved, although long‐term neurological sequelae may persist in patients with CNS involvement [13, 92].

### 6.6. Blastomyces

#### 6.6.1. Epidemiology

Blastomyces is a potentially life‐threatening opportunistic pathogen in immunocompromised children, with a significantly higher risk in those with impaired cell‐mediated immunity, such as T‐cell lymphopenia [89, 92–94]. This agent can also infect immunocompetent hosts, with manifestations ranging from asymptomatic infection to acute pneumonia, which is often clinically indistinguishable from bacterial pneumonia, acute respiratory distress syndrome (ARDS), or even death [92–94]. In patients with hematologic malignancies, the presentation is primarily severe pulmonary disease. After inhalation, the environmental conidia transform into a pathogenic broad‐based budding yeast phase at physiological temperatures, exhibiting increased virulence in the absence of functional T‐cells. Emerging species, such as Blastomyces helicus, pose additional challenges in diagnosis and treatment due to their distinct microbiological and clinical properties [93, 94].

#### 6.6.2. Clinical Manifestations

Acute pulmonary blastomycosis in immunocompromised children typically presents with a sudden onset of rapidly progressive pneumonia. This may mimic bacterial or other fungal infections, characterized by high fever, productive cough with purulent or hemoptic sputum, pleuritic chest pain, and worsening dyspnea that can progress to respiratory failure within hours. Constitutional symptoms, including weight loss, night sweats, and malaise, are also commonly observed. Severe pneumonia is the most frequent manifestation of blastomycosis in pediatric cases associated with hematologic malignancies, often progressing to ARDS and requiring mechanical ventilation. Chronic pulmonary blastomycosis develops over months, presenting with a persistent cough, low‐grade fever, and chest pain, which may be mistaken for other chronic infections or a relapse of the primary malignancy. Disseminated blastomycosis in immunocompromised hosts frequently involves the skin, bones, joints, CNS, and the genitourinary system [93, 94].

#### 6.6.3. Diagnostic Approach

The diagnosis of blastomycosis follows the EORTC/MSGERC criteria: possible, defined by clinical respiratory symptoms such as cough or dyspnea associated with nonspecific radiographic findings; probable, involving a positive antigen test in urine or serum, serology, or sputum culture, associated with clinical and radiological findings; and proven, requiring histopathological evidence of characteristic yeast forms or a positive culture from sterile tissue [22, 41]. Tissue biopsy and culture remain the gold standard; however, their invasive nature and the fact that cultures may take several weeks for confirmation make antigen detection or serological assays the preferred rapid alternatives. Radiographic findings are typically nonspecific, often demonstrating diffuse infiltrates or dense consolidation [93–95].

Urine and serum antigen tests are rapid and show significantly higher sensitivity in disseminated disease compared to localized pulmonary infection; however, they exhibit cross‐reactivity with other dimorphic fungi, particularly histoplasma [96–98]. Serological methods, such as complement fixation and immunodiffusion, have highly variable sensitivity and low specificity due to cross‐reactivity [97]. Fungal cultures are highly specific but slow growing, requiring specialized mycological expertise for the identification of both established and emerging species like B. helicus. Direct microscopic examination can be highly suggestive if the characteristic broad‐based budding yeasts are visualized. Histopathological examination using GMS or PAS staining typically reveals these yeast forms within areas of suppurative and granulomatous inflammation [97].

#### 6.6.4. Treatment and Outcomes

##### 6.6.4.1. Mild to Moderate Disease

Itraconazole is the primary antifungal agent for the treatment of stable, localized pulmonary blastomycosis, with a treatment duration typically spanning 6–12 months. Diligent TDM is strongly recommended to ensure that therapeutic serum concentrations are achieved and maintained [96, 98].

##### 6.6.4.2. Severe or Life‐Threatening Disease

For severe, life‐threatening infections, or in immunocompromised hosts presenting with respiratory failure and/or disseminated disease, liposomal amphotericin B (3–5 mg/kg/day) is the treatment of choice. This induction phase should continue until clinical stability is achieved (generally 1–2 weeks), followed by step‐down therapy with itraconazole for 12 months or until immune reconstitution [96, 98].

#### 6.6.5. CNS Involvement

In cases with CNS involvement, high‐dose liposomal amphotericin B (5 mg/kg/day) is recommended for 4–6 weeks. This should be followed by prolonged oral antifungal therapy, with serial monitoring to document the sterilization of the CSF. For severe pulmonary blastomycosis causing significant respiratory distress, a short course of systemic glucocorticoids may be considered to reduce the inflammatory response. If itraconazole is not tolerated or treatment failure occurs, voriconazole or posaconazole may be used as alternatives, although clinical experience with these agents in the pediatric population remains limited [97].

#### 6.6.6. Prophylaxis and Monitoring

Itraconazole prophylaxis may be considered in patients at exceptionally high risk within endemic regions, specifically those with severe T‐cell deficiencies or a history of previous blastomycosis. However, routine prophylaxis is not currently recommended due to the lack of established pediatric guidelines and the high risk of drug–drug interactions with complex chemotherapy regimens. Treatment efficacy is evaluated through clinical improvement, resolution of radiographic abnormalities, and a decrease in antigen titers; the latter serves as a reliable marker of therapeutic success [96, 97]. Survival rates in pulmonary blastomycosis vary significantly based on the severity of the underlying disease and immune status. Patients requiring mechanical ventilation due to ARDS face a notably higher risk of mortality, underscoring the necessity of timely diagnosis. Furthermore, survivors of severe disease may suffer from long‐term pulmonary function abnormalities [94, 97].

### 6.7. Cryptococcus

#### 6.7.1. Epidemiology and Species Distribution

Cryptococcus, encompassing Cryptococcus neoformans and Cryptococcus gattii, is an uncommon but potentially life‐threatening opportunistic fungal pathogen in immunocompromised children [99–103]. It particularly affects patients with hematologic malignancies or those undergoing HSCT, with a significant risk associated with T‐cell lymphopenia and GVHD. Distinct from many other opportunistic molds, Cryptococcus has a high predilection for the CNS, commonly causing meningitis, though it can also lead to primary pulmonary cryptococcosis in severely immunocompromised individuals. Its polysaccharide capsule is a major virulence factor, contributing to antiphagocytic activity and neurotropism. Primary risk factors in the pediatric population include profound lymphopenia, high‐dose corticosteroid exposure, severe GVHD, and significant defects in cellular immunity [104, 105].

#### 6.7.2. Clinical Manifestations

Pulmonary cryptococcosis typically presents with cough, dyspnea, fever, and pleuritic chest pain. While the infection may be asymptomatic or mild in immunocompetent or mildly immunocompromised hosts, it can vary significantly in severity among those with profound immunosuppression [104–106]. Manifestations range from incidental pulmonary nodules detected on routine imaging to rapidly progressive respiratory failure. Symptoms often include a dry cough that may become productive, chest discomfort, and fever with chills. Constitutional symptoms such as weight loss, night sweats, and generalized weakness may develop insidiously. If left untreated, pulmonary involvement in high‐risk patients frequently progresses to disseminated disease, most notably CNS infection [107–110].

Cryptococcal meningitis often presents as a subacute or chronic meningitis with an insidious onset of headache, altered mental status, fever, and nuchal rigidity. Focal neurological signs, such as cranial nerve palsies, seizures, and papilledema due to increased intracranial pressure (ICP), may also occur. Unlike acute bacterial meningitis, the symptoms of cryptococcal meningitis are often subtle and may be mistakenly attributed to the underlying malignancy or treatment side effects. Disseminated cryptococcosis can affect virtually any organ system; skin lesions, bone and joint involvement, or hepatosplenic lesions frequently serve as important diagnostic clues [107–110].

#### 6.7.3. Diagnostic Approach

The diagnosis of cryptococcosis follows the EORTC/MSGERC criteria: possible, characterized by clinical symptoms such as cough or fever with nonspecific radiological findings; probable, involving positive cryptococcal antigen in serum, CSF, BAL fluid, or sputum associated with clinical and radiological evidence; and proven, requiring histopathological evidence of encapsulated yeasts or a positive culture from sterile sites [22, 41, 104]. While tissue biopsy is the definitive gold standard, its invasive nature often limits its use in pediatric patients. In contrast, the cryptococcal antigen (CrAg) test is highly sensitive and specific, serving as a rapid diagnostic tool. The diagnostic yield of BAL or sputum culture is generally lower than that of antigen testing. Radiological findings are nonspecific and may include solitary or multiple nodules, consolidation, or cavitary lesions. Serum CrAg is highly accurate in detecting disseminated disease and correlates with the overall fungal burden and clinical response; notably, high initial titers are associated with a greater risk of therapeutic failure. In cases of CNS involvement, CSF antigen levels exhibit a similar correlation [110].

In patients with CNS cryptococcosis, lumbar puncture typically reveals increased opening pressure, elevated protein levels, and decreased glucose. Lymphocytic pleocytosis is also characteristic, though often less pronounced in severely immunosuppressed individuals. India ink staining remains a valuable rapid diagnostic tool, identifying encapsulated yeasts in a high proportion of CSF samples. While blood cultures are positive in most disseminated cases, the organism typically requires several days to grow. Although BAL cultures show lower sensitivity, diagnosis can often be achieved through CrAg testing and direct microscopic examination of respiratory specimens [104, 107].

#### 6.7.4. Treatment Protocols

In patients with pulmonary cryptococcosis lacking CNS involvement, fluconazole remains the treatment of choice for mild‐to‐moderate, localized infection in stable individuals, typically administered for 6–12 months. For severe disease, the recommended induction therapy consists of liposomal amphotericin B (3–5 mg/kg daily) combined with flucytosine for 2–4 weeks, followed by a 6–12‐month maintenance phase of fluconazole [110].

#### 6.7.5. CNS Cryptococcosis

Induction therapy consists of liposomal amphotericin B (3–5 mg/kg daily) combined with flucytosine for at least 4 weeks, a duration that may be extended based on clinical severity and CSF clearance. This is followed by consolidation therapy using fluconazole (6–12 mg/kg/day, maximum 400–800 mg/day, with appropriate pediatric dose adjustments) for 8–10 weeks, contingent upon documented CSF sterilization. Subsequently, maintenance therapy with fluconazole (3–6 mg/kg/day, maximum 200–400 mg/day) is continued until immune recovery, typically for up to 12 months. While voriconazole or posaconazole may serve as salvage options for patients intolerant to fluconazole, clinical experience with these agents in pediatric populations remains limited [107, 110]. Management of elevated ICP necessitates serial lumbar punctures or temporary CSF drainage for symptomatic patients. Although the use of corticosteroids in cryptococcal meningoencephalitis remains controversial, a short course may be considered in cases with a marked inflammatory response. Therapeutic success is monitored through clinical improvement, declining CrAg titers, and CSF sterilization; conversely, rising CrAg titers often suggest treatment failure. Effective response is further evidenced by the normalization of CSF cell counts and biochemical parameters [107, 110]. Primary prophylaxis may be considered for patients at high risk, particularly those with severe T‐cell dysfunction, despite limited pediatric data. In contrast, secondary prophylaxis is essential for chronically immunosuppressed patients with a history of cryptococcosis [111–113]. While overall survival rates are favorable when antifungal therapy succeeds, especially in cases of isolated pulmonary disease, neurological sequelae such as intellectual impairment, hearing loss, and motor deficits may persist in survivors of CNS involvement [107, 110].

### 6.8. Fusariosis

#### 6.8.1. Epidemiology and Emerging Importance

Fusarium species cause fusariosis, an opportunistic fungal infection primarily affecting immunocompromised pediatric patients, most notably neutropenic children with hematologic malignancies [4, 114]. The increasing incidence is attributed to improved diagnostic performance, a rising trend in voriconazole prophylaxis, which is ineffective against Fusarium species, and improved survival rates among severely immunocompromised individuals. Fusarium species are soil saprophytes and plant pathogens characterized by striking environmental persistence and resistance to routine antifungal drugs. Major risk factors in immunocompromised children include severe and prolonged neutropenia, severe mucositis, the presence of CVCs, and prior exposure to broad‐spectrum antifungal agents, particularly voriconazole. Unlike many other molds, Fusarium can affect both neutropenic and non‐neutropenic individuals, although the most severe outcomes are associated with profound neutropenia [4, 114].

#### 6.8.2. Clinical Manifestations and Diagnosis

Disseminated fusariosis is the most common presentation in severely immunocompromised children [4]. Clinical manifestations include fever, cough, and dyspnea, often presenting as disseminated disease with pulmonary involvement that mimics invasive aspergillosis.

Fusarium exhibits a unique tropism for the skin and subcutaneous tissues, forming characteristic painful subcutaneous nodules with central necrosis that may evolve into severe cellulitis. Pulmonary involvement is frequent in disseminated infections; symptoms include sustained fever despite broad‐spectrum antibiotic therapy, a productive cough that may progress to hemoptysis, pleuritic chest pain, and rapid progression to respiratory failure. Skin findings are invaluable for diagnosis; they typically manifest as erythematous papules or nodules that rapidly evolve into central necrosis or ulcerations, potentially mimicking cellulitis, drug reactions, or GVHD patterns. Localized infections may also involve the sinuses, eyes, and soft tissues, usually following trauma or invasive procedures. Fungemia is relatively common in disseminated cases; therefore, blood cultures are essential for diagnosis [114].

According to EORTC/MSGERC guidelines, the diagnosis is classified as possible, based on clinical and radiological signs and the presence of nodules; probably, characterized by a positive culture or PCR from BAL; or proven, requiring histopathologic confirmation of hyphae in sterile tissue [22, 41]. Radiological features are nonspecific and often identical to those of aspergillosis. Notably, blood cultures are positive in a significant number of patients with disseminated infections, providing a specific diagnostic criterion that is often absent in other mold infections. While the organism typically requires a few days to grow and may be confused with other fungi, a biopsy of skin lesions can rapidly ascertain the diagnosis via histopathology and culture. Histopathology reveals hyphae resembling *Aspergillus*, but Fusarium can be distinguished through culture morphology and molecular techniques. GM assays are usually negative in fusariosis, limiting their diagnostic utility. While PCR has shown potential, it requires further validation in the pediatric setting [115].

#### 6.8.3. Treatment and Prevention

Fusariosis is highly challenging due to its intrinsic resistance to various classes of antifungals. Fusarium species exhibit intrinsic resistance to echinocandins and most azoles; however, voriconazole and posaconazole retain variable activity against certain species; particularly, F. solani complex shows reduced susceptibility to all agents. These organisms frequently show reduced susceptibility to amphotericin B as well. The mainstay of treatment for fusariosis is liposomal amphotericin B at a dose of 5–10 mg/kg/day. Its antifungal efficacy against Fusarium species is superior to that of azoles. Liposomal amphotericin B is the most consistently effective antifungal agent against Fusarium species, although treatment response rates remain suboptimal even at maximally achievable doses. Combination therapy offers potential synergistic antifungal activity and broader coverage, particularly in disseminated infections or when initial monotherapy fails. Posaconazole demonstrates enhanced activity against certain Fusarium species compared to other azoles and may serve as salvage therapy or step‐down treatment for patients showing clinical improvement with amphotericin B; however, TDM is essential [116].

Surgical intervention is critical, with intensive debridement of infected lesions being highly effective. Total resection of accessible lesions, in conjunction with antifungal therapy, is more effective than antifungal therapy alone in improving survival outcomes. Immune recovery is the most significant prognostic factor; specifically, neutrophil recovery and the reduction of immunosuppression are essential for successful treatment [115, 116].

#### 6.8.4. Antifungal Prophylaxis

Antifungal prophylaxis in pediatric patients with hematologic malignancies and those undergoing HSCT requires meticulous risk stratification based on the underlying disease, treatment intensity, duration of immunosuppression, and local epidemiological factors. Evidence‐based guidelines emphasize that prophylactic strategies must be tailored to specific patient populations, as universal approaches are often ineffective and may promote the emergence of resistant organisms or lead to unnecessary toxicity [117]. Table 2 presents a practical framework for antifungal prophylaxis in pediatric patients, categorized by risk level.

**TABLE 2:** Risk‐stratified antifungal prophylaxis framework for pediatric patients.

Risk category	Patient population	IFI risk (%)	Primary pathogens	Evidence‐based prophylaxis	Evidence quality	Clinical considerations
Very High Risk	AML (all phases)	23.1 [28]	*Aspergillus*, *Candida*, Mucorales	Posaconazole/Voriconazole	High [111, 112]	Continue through neutrophil recovery
Very High Risk	Allogeneic HSCT	16.9 [28]	*Aspergillus*, *Candida*	Mold‐active agents	High [41, 111]	Pre‐engraftment period critical
Very High Risk	Relapsed/Refractory ALL	Substantially elevated	*Aspergillus*, *Candida*	TMP–SMX + Mold‐active	Moderate [41]	Intensified salvage regimens
Moderate Risk	ALL (de novo)	12.1 [28]	*Pneumocystis jirovecii*	TMP–SMX only	High [41, 111]	Throughout treatment
Moderate Risk	High‐grade lymphomas	Variable	*Aspergillus*, *Candida*	Risk‐based approach	Moderate [41]	Individualized assessment
Lower Risk	Autologous HSCT	4.0 [28]	*Candida* spp.	Fluconazole	Moderate [41, 111]	Through neutrophil recovery
Lower Risk	Solid tumors	1.8 [28]	*Candida* spp.	Risk‐based approach	Low [41]	Limited benefit in most cases

*Note:* TMP–SMX, trimethoprim–sulfamethoxazole.

Abbreviations: ALL, acute lymphoblastic leukemia; AML, acute myeloid leukemia; IFI, invasive fungal infection; HSCT, hematopoietic stem cell transplantation.

## 7. Prophylaxis Strategies Based on Specific Disease

### 7.1. Acute Lymphoblastic Leukemia

For standard‐risk de novo ALL, primary prophylaxis with TMP–SMX for PJP prevention is recommended. Routine mold‐active antifungal prophylaxis is not suggested for standard‐risk patients, as the moderate baseline fungal infection risk is adequately addressed with TMP–SMX prophylaxis, and concerns exist regarding the selection of resistant organisms. Prophylaxis duration continues throughout the treatment [111, 112].

### 7.2. Acute Myeloid Leukemia

Primary prophylaxis consisting of posaconazole or voriconazole is recommended for all AML patients. The high baseline fungal infection risk (23.1%) and prolonged neutropenia mandate mold‐active prophylaxis. Duration extends from the start of induction through neutrophil recovery. Micafungin serves as an alternative when azoles are contraindicated [28, 41, 111, 112].

For high‐risk or relapsed AML, primary prophylaxis with TMP–SMX plus a mold‐active agent (e.g., posaconazole with food) during intensive phases is suggested. The significantly elevated fungal infection risk justifies mold‐active prophylaxis. The duration covers periods of expected prolonged neutropenia [41, 111, 112].

### 7.3. HSCT

For allogeneic transplantation during the pre‐engraftment phase, primary prophylaxis consisting of posaconazole or voriconazole is recommended. Micafungin is an alternative option for patients with azole intolerance or significant drug interactions. The duration extends from the start of the conditioning regimen until neutrophil engraftment. For patients in the post‐engraftment phase following allogeneic transplantation, antifungal prophylaxis may be discontinued if no GVHD exists and immunosuppression has ceased. Patients with GVHD should continue posaconazole or switch to an alternative based on disease severity and steroid requirements. Duration continues until GVHD resolution and the cessation of immunosuppressive therapy [41, 111, 112].

In autologous transplantation patients, primary prophylaxis includes fluconazole for yeast. Enhanced prophylaxis with mold‐active agents should be considered for patients with prior fungal infections or extended conditioning regimens. Duration extends through neutrophil recovery. Analysis demonstrates that IFIs are rare in pediatric and young adult autologous transplantation patients, occurring in less than 2% of cases [28, 41, 111, 112].

### 7.4. Agent‐Specific Considerations

The primary antifungal agents utilized for prophylaxis, including their respective advantages, challenges, monitoring requirements, and pediatric‐specific considerations, are summarized in Table 3.

**TABLE 3:** Antifungal prophylaxis agents: Characteristics and monitoring.

Agent	Advantages	Challenges	Monitoring requirements	Pediatric considerations
Posaconazole [111, 112]	Broad‐spectrum including Mucorales	Variable bioavailability, drug interactions	Serum level monitoring recommended	Delayed‐release tablets preferred
Voriconazole [111, 112]	Excellent bioavailability, IV/PO	Hepatotoxicity, photosensitivity	Trough levels essential	Higher dosing due to increased clearance
Micafungin [41]	Excellent safety, no drug interactions	Limited mold coverage, IV only	Generally unnecessary	Well tolerated in different ages
Fluconazole [112, 113]	Excellent safety, low cost	No mold activity	Unnecessary except for renal impairment	Limited to low‐risk patients

*Note:* IV, intravenous; PO, per os (oral).

## 8. Breakthrough Infections and Economic Considerations

Despite optimal prophylaxis, breakthrough IFIs can occur in high‐risk patients, necessitating robust surveillance strategies that include regular clinical assessments and biomarker monitoring (e.g., GM, β‐D‐glucan) [41, 112]. Key resistance considerations include Mucorales breakthrough during azole prophylaxis and the emergence of azole‐resistant *Aspergillus*. Management requires prompt diagnostic evaluation, escalation of empirical therapy, and modification of the prophylactic regimen. Evidence‐based implementation mandates a regular review of institutional fungal epidemiology and resistance patterns, multidisciplinary consensus on prophylaxis protocols, staff education regarding appropriate prescribing and monitoring, and systematic outcome tracking [41, 111].

### 8.1. Recent Advances

Recent diagnostic advancements have significantly enhanced the detection and management of fungal infections in children. NGS, particularly metagenomic sequencing of pediatric BAL specimens, enables the rapid identification of novel, emerging, and coinfecting fungal species, as well as antifungal resistance genes [28]. Meanwhile, artificial intelligence (AI) tools are increasingly integrated into clinical practice. Machine learning models utilize electronic health records (EHRs) to facilitate risk stratification, while deep learning techniques improve the early detection of fungal infections on chest imaging. Furthermore, natural language processing (NLP) is employed to identify high‐risk patients in real time. Additionally, AI‐based drug interaction models are being utilized to precision‐tailor antifungal therapy [118].

## 9. Future Directions

Future objectives include developing rapid point‐of‐care tests that combine multiple biomarkers to facilitate real‐time clinical decision‐making, alongside standardizing pediatric NGS procedures for various specimen types. Enhancing multiplex PCR panels and identifying age‐specific biomarkers are essential for precise risk assessment, accounting for the differences in biomarker dynamics between children and adults. Therapeutic advancements are expected from pharmacokinetic and toxicological evaluations of new antifungals specific to pediatric patients, the optimization of drug combinations, and the development of immunotherapies such as T cells specific to pathogens, therapeutic vaccines, and immunomodulatory agents. These approaches must carefully balance antifungal efficacy against the risks of malignancy progression and GVHD [119].

The field could also benefit from integrating pharmacogenetics with machine learning tools to refine the early diagnosis and prevention of IFIs [118]. However, pediatric‐specific prediction models must be validated across diverse populations. Moreover, research evaluating cost‐effective and evidence‐based antifungal stewardship models, particularly in resource‐limited settings, is highly recommended. Finally, the establishment of global collaborative research networks is crucial for improving clinical outcomes for pediatric patients worldwide.

## 10. Conclusion

This study addresses critical scientific gaps in the management of pediatric fungal infections by providing an evidence‐based framework for risk stratification, diagnosis, and treatment. Risk prediction models have demonstrated superior predictive capabilities compared to clinical judgment alone. Furthermore, this study evaluated the performance characteristics of diagnostic tests specifically within pediatric populations, revealing that multimodal diagnostic approaches achieve enhanced accuracy. These findings offer practical guidance to improve clinical outcomes and the quality of life for high‐risk patients while optimizing the utilization of healthcare resources.

## Funding

There is no fund available for this research.

## Ethics Statement

The study protocol was approved by the Research Ethics Committee of the Shahid Sadoughi University of Medical Sciences (IR. SSU. REC.1404.027).

## Conflicts of Interest

The authors declare no conflicts of interest.

## Data Availability

Data sharing is not applicable to this article.
